# Real-world comorbidities and treatment patterns of patients with acromegaly in two large US health plan databases

**DOI:** 10.1007/s11102-012-0432-6

**Published:** 2012-10-11

**Authors:** Tanya Burton, Elisabeth Le Nestour, Tim Bancroft, Maureen Neary

**Affiliations:** 1 Health Economics and Outcomes Research, OptumInsight, Waltham, MA 02493 USA; 2 PharmaNet/i3, Nanterre, France; 3 Novartis Pharmaceuticals Corporation, East Hanover, NJ USA; 4 Health Economics and Outcomes Research, OptumInsight, Eden Prairie, MN 55344 USA

**Keywords:** Acromegaly, Treatment patterns, Comorbidities, Somatostatin analogues, Octreotide, Lanreotide, Pegvisomant, Bromocriptine, Cabergoline

## Abstract

Acromegaly is a rare, chronic, and debilitating disease that results from excessive growth hormone production. Clinically, this disease is associated with enlargement of soft tissue, excessive skeletal growth, and increased risk of cardiovascular disease. Acromegaly is often diagnosed late, when a wide range of comorbidities may already be present. First-line therapy for acromegaly is typically surgery; but a number of highly-specific pharmacological agents have recently enabled a more aggressive medical management of acromegaly. Since surgical cure of acromegaly is low for macroadenomas, medical control of active acromegaly is an important component of treatment. There are no published US data currently available regarding real-world rates of comorbidities and treatment patterns among patients with acromegaly. This retrospective study examined the comorbidities and treatment patterns of 949 health plan enrollees, who had acromegaly diagnosis and/or procedure codes in an administrative claims database from July 1, 2002 through June 30, 2010. Acromegaly was associated with high rates of hypertension and diabetes along with a number of other comorbidities. The incidence of comorbidities was highest among patients with acromegaly-related treatment, which may have resulted, in part, from inadequate disease management and/or poor disease control. Unexpectedly, 55 % of patients identified with acromegaly received no treatment for acromegaly (i.e., surgery, radiotherapy, and medication) and only 28 % received a medication treatment during the observation period. However, some patients may have received a curative surgery prior to the observation period, which may have reduced the use of other acromegaly-related treatments during the study period. Of those treated with medications, the most common first medications were octreotide, cabergoline, and bromocriptine. Given the high incidence of serious comorbidities associated with active acromegaly, earlier diagnosis and treatment, along with appropriate follow-up care, may potentially avoid the life-long consequences of uncontrolled disease.

## Introduction

Acromegaly is a debilitating disorder caused by the chronic hypersecretion of growth hormone (GH). The most frequent source of excessive GH is a benign pituitary adenoma, which progresses slowly and may remain undetected for up to 10 years [1]. The risk of morbidity and mortality increases as the tumor size grows and GH hypersecretion continues [2, 3]. Patients often present with arrhythmias, hypertension, and valvular heart disease by the time of acromegaly diagnosis [4]. Acromegaly treatments aim to reduce or control tumor growth, decrease GH levels, and normalize insulin-like growth factor 1 (IGF-1) concentrations, a hormone stimulated by GH production [5]. Certain clinical manifestations of acromegaly can be reversed or slowed with treatment [1, 2, 5–7]. Some reversible conditions include carpal tunnel syndrome, sweating, and obstructive sleep apnea, but others such as diabetes, hypertension, and heart disease may remain the same or only show slight improvement with acromegaly treatment [8]. By correcting the overproduction of GH and IGF-1, a recent meta-analysis suggests that the mortality for treated acromegaly patients was nearly equivalent to the mortality expected of the general population [9].

The primary treatment for acromegaly is tumor excision via transsphenoidal surgery. At diagnosis, more than 75 % of acromegaly patients have a macroadenoma (>10 mm in diameter) but <50 % of macroadenomas are controlled with surgery alone [4, 6]. Among acromegaly patients with a microadenoma, surgery is more than 80 % effective at lowering GH and normalizing IGF-1 [4]. Post-operative treatments consist of medical therapy and radiotherapy. Medical therapy for acromegaly includes medications in three drug classes: injectable somatostatin analogs [octreotide acetate, octreotide long-acting release (LAR), and lanreotide]; injectable GH receptor antagonist (pegvisomant); and oral dopamine agonists (bromocriptine and cabergoline). Medical therapy may be given as a first-line therapy when surgery is too dangerous or unlikely to cure or as a second-line therapy if the tumor recurred or surgery failed to achieve biochemical control [5]. Up to 10 % of removed tumors recur [4]. Radiotherapy is often given as a third-line therapy to patients unable to withstand medical therapy or as a second-line therapy to patients with a tumor inaccessible through surgery. However, radiotherapy is not as safe as some of the other therapy options. For patients receiving new or conventional radiotherapy, more than 50 % are at risk of hypopituitarism, and nearly 6 % are at risk of vision defects [5]. Secondary tumors and cerebrovascular events are also a potential risk with conventional radiotherapy.

Acromegaly is a rare condition with an estimated annual incidence of only 3.3 cases per million and a prevalence ranging from 40 to 130 per million [6, 10]. Data on the burden of illness for acromegaly patients are limited, and there are no published US data currently available. The goal of this study was to examine real-world comorbidities and treatment of acromegaly patients in US commercial health plans.

## Methods

### Study design

This retrospective study analyzed administrative claims from two large commercial health plan databases from July 1, 2002 through June 30, 2010. The Impact National Benchmark Database™ and the Life Sciences Research Database, both affiliated with OptumInsight (formerly Innovus), contain medical and pharmacy claims, and enrollment information from more than 40 US health plans with over 14 million and 89 million health plan enrollees, respectively. Health plans included in these databases were geographically diverse across the US.

Medical (professional and facility) claims included *International Classification of Diseases, 9th Revision, Clinical Modification* (ICD-9-CM) diagnosis codes, ICD-9-CM procedure codes, *Current Procedural Terminology, Version 4* (CPT-4) procedure codes, *Healthcare Common Procedure Coding System* (HCPCS) procedure codes, site of service codes, and health plan and patient paid amounts. Outpatient pharmacy claims included national drug codes (NDC) for dispensed medications, dosage form, fill date, health plan, and patient paid amounts. All administrative claims data were de-identified and compliant with the provisions of the Health Insurance Portability and Accountability Act of 1996.

### Study patient identification

Health plan enrollees were included in this analysis if they met one of the following three criteria to identify patients with acromegaly: (1) had at least two medical claims separated by at least 30 days with a primary or secondary acromegaly diagnosis code (ICD-9-CM: 253.0) between January 1, 2003 and June 30, 2009 (identification period), (2) had at least one inpatient facility claim with a primary or secondary acromegaly diagnosis code and an acromegaly-related procedure code (“Appendix”) during the identification period, or (3) had at least one claim of any type with an acromegaly-related procedure code followed by at least one medical claim from any place of service with a primary or secondary acromegaly diagnosis code during the identification period. The index date was defined as the date of the earliest claim for an acromegaly-related diagnosis code, procedure code, or medication (i.e., octreotide acetate, octreotide LAR, lanreotide, pegvisomant, bromocriptine, cabergoline) during the identification period.

In addition to the criteria above, patients had to be continuously enrolled with medical and pharmacy benefits for 6 months before the index date (pre-index period) and until the earliest of: death (as evidenced by hospital discharge claims or Social Security Administration death records); disenrollment from the health plan; or the end of the study period on June 30, 2010 (follow-up period). Patients were assigned to one of four treatment status cohorts based on evidence of acromegaly-related surgical procedures (“Appendix”) and medication use during the study period: Surgery Only, Surgery + Meds, Meds Only, and None. Additionally, patients who received at least one acromegaly-related medication during the study period (i.e., patients in the Surgery + Meds and Meds Only cohorts) were assigned to one of six subgroups based on the first claim for an acromegaly-related medication on or after the index date. With respect to the two octreotide agents, octreotide acetate and LAR are considered the short- and long-acting formulations of the same medication.

### Study measures

The primary outcomes included incident cases of acromegaly-related comorbidities and complications (“Appendix”) during the follow-up period. The selected comorbidities and complications were derived from a list of acromegaly clinical features presented in Cordero et al. [11]. Incident cases were defined by the first occurrence of a claim for a comorbidity or complication during the follow-up period, given that no claims for the comorbidity or complication were present prior to the index date. The time at risk for a comorbidity or complication was calculated in days from the index date up until the date of the first claim for the comorbidity or complication.

Demographic variables were measured at the start of the pre-index period and included age, gender, and US census geographic regions of the health plans. Pre-index clinical characteristics included the Quan–Charlson comorbidity score [12]. All-cause death during the follow-up period was also reported.

### Analyses

All study variables were analyzed descriptively by treatment status cohorts. Categorical variables were summarized with frequencies and percentages and continuous variables were summarized with means and standard deviations (SD). Traditional *F-* tests were performed to assess cohort differences in the continuous measures, and depending on the sample size, either χ^2^-tests or Fisher’s exact tests were performed to assess cohort differences in the categorical measures.

Counts of acromegaly-related comorbidities and complications during the follow-up period were tabulated by study cohort. Incidence rates were calculated for each comorbidity and complication as the number of new cases divided by the observed person-days at risk (expressed as per 10,000 person-days). Pairwise cohort comparisons of each comorbidity and complication were assessed using unadjusted incidence rate ratios (IRR) and the exact binomial test. All analyses were performed using SAS v9.2 (SAS Institute, Cary, NC, USA). A *p* value <0.05 was used to signify statistical significance.

## Results

### Baseline characteristics

Of the nearly 37 million commercial health plan enrollees in the databases between January 1, 2003 and June 30, 2009, 54,413 had at least one claim with an acromegaly diagnosis code, procedure code, or medication; of which 949 met the remaining study inclusion criteria. The date of the earliest acromegaly claim (i.e., the index date) was based on a claim with an acromegaly diagnosis code or procedure code for 94 % of the study patients. For the remaining 6 %, the index date was based on a claim for an acromegaly-related medication. However, all study patients had the required acromegaly diagnosis codes and procedure codes necessary for study entry some time during the follow-up period.

Table 1 displays the baseline demographic and clinical characteristics of the total study sample and treatment status cohorts. Of the 949 patients identified with acromegaly, 162 (17 %) had evidence of an acromegaly-related surgical procedure only (Surgery Only cohort), 140 (15 %) had evidence of an acromegaly-related surgical procedure and medication (Surgery + Meds cohort), 123 (13 %) had evidence of an acromegaly-related medication only (Meds Only cohort), and 524 (55 %) had no evidence of a surgical procedure or medication (None cohort) during the follow-up period. Overall, patients had a mean (SD) age of 42 (18) years and 52 % were male at the time of the earliest acromegaly claim. Patients in the Surgery Only cohort were on average older (48 years) than the other study cohorts. All cohorts were comparable with respect to gender. The greatest geographic representation overall, and within each cohort, came from Southern (39 % overall) and Northeastern (32 % overall) US census regions. The majority of patients had a Quan–Charlson score <2; the Surgery Only and Meds Only cohorts had the highest proportion of patients with a score of 2 or more.

**Table 1:** Baseline demographic and clinical characteristics

	Total (*N* = 949)	(1) Surgery only (*N* = 162)	(2) Surgery and medication (*N* = 140)	(3) Medication only (*N* = 123)	(4) Neither (*N* = 524)	(1) versus (2) *p* value	(1) versus (3) *p* value	(1) versus (4) *p* value	(2) versus (3) *p* value	(2) versus (4) *p* value	(3) versus (4) *p* value
Age, years, mean (SD)	42.1 (17.8)	48.0 (11.6)	41.8 (12.6)	45.7 (14.5)	39.5 (20.4)	<0.001	0.147	<0.001	0.020	0.101	<0.001
Male, *n* (%)	489 (51.5)	87 (53.7)	68 (48.6)	65 (52.8)	269 (51.3)	0.374	0.886	0.598	0.489	0.561	0.763
Geographic region, *n* (%)											
Northeast	301 (31.7)	49 (30.2)	52 (37.1)	32 (26.0)	168 (32.1)	0.205	0.433	0.664	0.053	0.256	0.192
Midwest	189 (19.9)	29 (17.9)	39 (27.9)	24 (19.5)	97 (18.5)	0.039	0.729	0.861	0.114	0.015	0.798
South	368 (38.8)	62 (38.3)	41 (29.3)	58 (47.2)	207 (39.5)	0.100	0.132	0.779	0.003	0.026	0.120
West	91 (9.6)	22 (13.6)	8 (5.7)	9 (7.3)	52 (9.9)	0.023	0.093	0.190	0.598	0.123	0.373
Quan–Charlson comorbidity score, *n* (%)											
Low (≤1)	786 (82.8)	119 (73.5)	120 (85.7)	94 (76.4)	453 (86.5)	0.009	0.568	<0.001	0.053	0.822	0.006
High (≥2)	163 (17.2)	43 (26.5)	20 (14.3)	29 (23.6)	71 (13.5)						
Length of follow-up, days, mean (SD)	970.9 (655.0)	780.9 (497.3)	1,096.4 (711.3)	1,024.0 (743.2)	983.6 (649.6)	<0.001	0.002	<0.001	0.421	0.074	0.579
Follow-up all-cause death, *n* (%)	10 (1.1)	3 (1.9)	1 (0.7)	1 (0.8)	5 (1.0)	0.389	0.460	0.352	0.927	0.790	0.883

Overall, the average ± SD length of follow-up was 971 ± 655 days, with a maximum of 2,737 days or 7.5 years. The follow-up times for the Surgery + Meds (1,096 ± 711 days), Meds only (1,024 ± 743 days), and None (984 ± 650 days) cohorts were similar (Surgery + Meds vs. Meds only: *p* = 0.421; Surgery + Meds vs. None: *p* = 0.074; Meds only + None: *p* = 0.579), but they were all longer compared to the Surgery only cohort (781 ± 497 days; all *p* < 0.001). The overall proportion of death was small (1 %) and the difference was not statistically significant across cohorts.

### Incidence of acromegaly-related comorbidities and complications

Table 2 shows the incidence and incidence rate ratios of 23 acromegaly-related comorbidities and complications. The highest incidence rates overall were for arthropathy/arthralgia/synovitis (4.5 per 10,000 person-days) and hypertension (3.9 per 10,000 person-days); followed by hypopituitarism (2.3 per 10,000 person-days), osteoarthritis (2.1 per 10,000 person-days), diabetes (1.9 per 10,000 person-days), colon polyp (1.6 per 10,000 person-days), myopathy/myalgia and cardiac dysrhythmia/arrhythmia (both 1.4 per 10,000 person-days), valvular heart disease and sleep apnea (both 1.3 per 10,000 person-days), and menstrual abnormality (1.1 per 10,000 person-days). Among these, the greatest observed differences were between the treated (Surgery Only, Surgery + Meds, and Meds Only) and untreated (None) cohorts.

**Table 2:** Incidence rate (IR) per 10,000 person-years and incidence rate ratio (IRR) of acromegaly-related comorbidities and complications by treatment status cohorts

	Total (*N* = 949)	(1) Surgery only (*N* = 162)	(2) Surgery +meds (*N* = 140)	(3) Meds only (*N* = 123)	(4) None (*N* = 524)	(1 vs. 2) IRR	(1 vs. 3) IRR	(1 vs. 4) IRR	(2 vs. 3) IRR	(2 vs. 4) IRR	(3 vs. 4) IRR
Local effects, *n* [IR]											
Visual-field defects	55 [0.644]	16 [1.462]	21 [1.676]	4 [0.340]	14 [0.279]	0.872	4.301**	5.241***	4.931***	6.009***	1.219
Hypopituitarism	165 [2.267]	47 [5.037]	38 [3.528]	27 [2.966]	53 [1.216]	1.428	1.698*	4.141***	1.189	2.901***	2.439***
Musculoskeletal, *n* [IR]											
Osteoarthritis	154 [2.062]	25 [2.460]	23 [1.792]	18 [1.723]	88 [2.134]	1.372	1.428	1.153	1.040	0.840	0.808
Arthropathy/arthralgia/synovitis	263 [4.503]	39 [4.773]	38 [3.852]	39 [4.801]	147 [4.558]	1.239	0.994	1.047	0.802	0.845	1.053
Kyphosis and scoliosis	28 [0.316]	2 [0.160]	2 [0.134]	5 [0.420]	19 [0.386]	1.190	0.380	0.415	0.320	0.348	1.090
Vertebral fracture	13 [0.144]	1 [0.079]	2 [0.133]	2 [0.162]	8 [0.159]	0.596	0.491	0.501	0.824	0.840	1.019
Carpal tunnel syndrome	37 [0.424]	10 [0.914]	6 [0.414]	8 [0.677]	13 [0.260]	2.208	1.348	3.509**	0.611	1.589	2.602*
Myopathy/myalgia	112 [1.424]	10 [0.864]	12 [0.909]	16 [1.474]	74 [1.720]	0.952	0.587	0.503*	0.617	0.528*	0.857
Cardiovascular, *n* [IR]											
Hypertension	207 [3.908]	39 [7.129]	35 [4.209]	30 [4.460]	103 [3.173]	1.694*	1.598	2.247***	0.944	1.327	1.406
Cardiomyopathy	23 [0.257]	6 [0.503]	4 [0.271]	4 [0.320]	9 [0.179]	1.854	1.571	2.811	0.847	1.516	1.789
Cardiac hypertrophy	47 [0.538]	8 [0.677]	3 [0.202]	6 [0.497]	30 [0.617]	3.349	1.360	1.097	0.407	0.328*	0.807
Heart failure	35 [0.396]	7 [0.590]	1 [0.067]	5 [0.416]	22 [0.443]	8.820*	1.419	1.333	0.161	0.151*	0.940
Valvular heart disease	110 [1.343]	23 [2.179]	20 [1.469]	20 [1.839]	47 [1.003]	1.483	1.184	2.171**	0.799	1.464	1.833*
Cardiac dysrhythmia/arrhythmia	110 [1.357]	23 [2.214]	23 [1.744]	14 [1.285]	50 [1.073]	1.269	1.723	2.064**	1.358	1.626	1.198
Respiratory, *n* [IR]											
Nasal polyps	18 [0.199]	2 [0.162]	5 [0.335]	3 [0.242]	8 [0.157]	0.485	0.671	1.032	1.385	2.128	1.537
Sleep apnea (obstructive, central)	101 [1.301]	20 [2.236]	24 [2.104]	14 [1.317]	43 [0.921]	1.063	1.698	2.427**	1.598	2.284**	1.429
Narcolepsy	2 [0.022]	1 [0.079]	0 [0.000]	0 [0.000]	1 [0.019]	–	–	4.071	–	0.000	0.000
Endocrine/metabolic, *n* [IR]											
Diabetes (incl. impaired glucose tolerance)	135 [1.931]	23 [2.958]	26 [2.370]	29 [3.422]	57 [1.335]	1.248	0.864	2.215**	0.692	1.775*	2.563***
Galactorrhea	6 [0.066]	0 [0.000]	0 [0.000]	3 [0.251]	3 [0.059]	–	0.000	0.000	0.000	0.000	4.281
Menstrual abnormality	89 [1.097]	12 [1.047]	15 [1.195]	12 [1.158]	50 [1.069]	0.876	0.904	0.979	1.032	1.117	1.083
Impaired libido/impotence	54 [0.631]	12 [1.031]	4 [0.274]	10 [0.867]	28 [0.586]	3.767**	1.189	1.758	0.316*	0.467	1.479
Other, *n* [IR]											
Colon polyp	125 [1.554]	27 [2.620]	26 [2.142]	20 [1.864]	52 [1.100]	1.223	1.405	2.383***	1.149	1.948**	1.695
Hyperhydrosis	16 [0.177]	2 [0.161]	2 [0.139]	2 [0.160]	10 [0.197]	1.161	1.003	0.818	0.864	0.705	0.816

* <0.05; ** <0.01; *** <0.001

Compared to the untreated cohort, all three treatment cohorts had significantly higher incidence rates of hypopituitarism (IRR: 4.1, 2.9, and 2.4, respectively, all *p* < 0.001) and diabetes (IRR: 2.2, 1.8, 2.6, all *p* < 0.05). The Surgery Only cohort had significantly higher rates of hypertension (IRR: 2.2; *p* < 0.001), valvular heart disease (IRR: 2.2; *p* < 0.01), cardiac dysrhythmia/arrhythmia (IRR: 2.1; *p* < 0.01), and sleep apnea (IRR: 2.4; *p* < 0.01) than the None cohort. The Surgery + Meds cohort had a significantly higher rate of sleep apnea (IRR: 2.3; *p* < 0.01) and the Meds Only cohort had a significantly higher rate of valvular heart disease (IRR: 1.8; *p* < 0.05) than the None cohort. The Surgery + Meds (IRR: 2.4; *p* < 0.001) and Meds Only (IRR: 1.9; *p* < 0.01) cohorts both had significantly higher rates of colon polyps than the None cohort. However, the reverse was found with myopathy/myalgia; the Surgery Only (IRR: 0.5; *p* < 0.05) and Surgery + Meds (IRR: 0.5; *p* < 0.05) cohorts had significantly lower rates of myopathy/myalgia than the None cohort. Among the treatment cohorts, the Surgery Only cohort was found to have a significantly higher rate of hypertension than the Surgery + Meds cohort (IRR: 1.7; *p* < 0.05).

While the incidence rates of these outcomes were very low, the Surgery Only cohort had significantly higher rates of heart failure (IRR: 8.8; *p* < 0.05) and impaired libido/impotence (IRR: 3.8; *p* < 0.01) compared to the Surgery + Meds cohort; the Surgery Only cohort also had a significantly higher rate of visual defects than the Meds Only cohort (IRR: 4.3; *p* < 0.01); while the Surgery + Meds cohort had a significantly higher rate of visual-field defects (IRR: 4.9; *p* < 0.001) but a significantly lower rate of impaired libido/impotence (IRR: 0.3; *p* < 0.05) than the Meds Only cohort.

Compared to the None cohort, remaining differences included: the Surgery Only cohort had higher rates of visual-field defects (IRR: 5.2; *p* < 0.001) and carpal tunnel syndrome (IRR: 3.5; *p* < 0.01); the Surgery + Meds cohort had lower rates of cardiac hypertrophy (IRR: 0.03; *p* < 0.05) and heart failure (IRR: 0.2; *p* < 0.05); and the Meds Only cohort had a higher rate of carpal tunnel syndrome (IRR: 2.6; *p* < 0.05).

### Medical therapy subanalysis

Use of an acromegaly-related medication during the follow-up period is presented in Table 3 for the 263 study patients in the Surgery + Meds and Meds Only cohorts. Nearly half (48.7 %) of the sample had at least one claim for octreotide acetate during the follow-up period. Other common follow-up medications included octreotide LAR (45.3 %) and cabergoline (32.7 %). Use of all medications, except lanreotide, did not differ between cohorts. A higher proportion of patients in the Surgery + Meds cohort were prescribed lanreotide compared to patients in the Meds Only cohort (11.4 vs. 4.1 %; *p* = 0.028). The mean (SD) time between the index date and the date for the first medication claim was 233 (339) days for the Surgery + Meds cohort and 119 (315) days for the Meds Only cohort.

**Table 3:** Follow-up acromegaly-related medication use

Acromegaly-related medication, *n* (%)	Total (*N* = 263)	Surgery + meds (*N* = 140)	Meds only (*N* = 123)	*p* value
Octreotide acetate	128 (48.7)	74 (52.9)	54 (43.9)	0.147
Octreotide LAR	119 (45.3)	66 (47.1)	53 (43.1)	0.510
Lanreotide	21 (8.0)	16 (11.4)	5 (4.1)	0.028
Pegvisomant	45 (17.1)	27 (19.3)	18 (14.6)	0.318
Bromocriptine	32 (12.2)	13 (9.3)	19 (15.4)	0.127
Cabergoline	86 (32.7)	46 (32.9)	40 (32.5)	0.956
Time to index medication, days, mean (SD)	179 (332)	233 (339)	119 (315)	0.005

Table 4 displays the subsequent medications that patients were prescribed following their first (index) medication. Of the 263 patients with at least one claim for an acromegaly-related medication during the follow-up period, 103 (39.2 %) had an index claim for octreotide acetate, 49 (18.6 %) for octreotide LAR, 11 (4.2 %) for lanreotide, 17 (6.5 %) for pegvisomant, 26 (9.9 %) for bromocriptine, and 57 (21.7 %) for cabergoline.

**Table 4:** Subsequent medication use following index acromegaly-related medication

Subsequent medication, *n* (%)	Index medication					
	Octreotide ACE (*N* = 103)	Octreotide LAR (*N* = 49)	Lanreotide (*N* = 11)	Pegvisomant (*N* = 17)	Bromocriptine (*N* = 26)	Cabergoline (*N* = 57)
Octreotide acetate (ACE)	–	3 (6.1)	0 (0.0)	3 (17.6)	4 (15.4)	15 (26.3)
Octreotide LAR	54 (52.4)	–	1 (9.1)	3 (17.6)	1 (3.8)	11 (19.3)
Lanreotide	3 (2.9)	4 (8.2)	–	0 (0.0)	0 (0.0)	3 (5.3)
Pegvisomant	17 (16.5)	3 (6.1)	2 (18.2)	–	1 (3.8)	5 (8.8)
Bromocriptine	4 (3.9)	1 (2.0)	0 (0.0)	0 (0.0)	–	1 (1.8)
Cabergoline	17 (16.5)	5 (10.2)	1 (9.1)	2 (11.8)	4 (15.4)	–

More than half (52.4 %) of the patients with an index octreotide acetate claim later filled a prescription for octreotide LAR. However, fewer patients added a medication in the other subgroups during the follow-up period. The most commonly added medication in each subgroup included: cabergoline was added by 5 (10.2 %) with an index octreotide LAR and 4 (15.4 %) with an index bromocriptine prescription; pegvisomant was added by 2 (18.2 %) with an index lanreotide prescription; octreotide acetate was added by 3 (17.6 %) with an index pegvisomant, 4 (15.4 %) with an index bromocriptine and 15 (26.3 %) with an index cabergoline prescription.

## Discussion

This retrospective analysis of 949 acromegaly patients was performed using administrative claims data from two large health plan databases in the United States. The study evaluated the incidence of comorbidities and complications and the use of acromegaly-related medications among acromegaly patients. Differences in these clinical outcomes were examined across treatment status cohorts. The results establish that for nearly half of all comorbidities and complications considered, the incidence was low (<1 per 10,000 person-days) and/or did not differ across treatment status cohorts. However, a number of major comorbidities were identified with higher frequency (≥1 per 10,000 person-days) such as arthropathy, hypertension, hypopituitarism, osteoarthritis, and diabetes. Other common conditions included: colon polyps, myopathy/myalgia, cardiac dysrhythmia/arrhythmia, valvular heart disease, sleep apnea, and menstrual abnormality. Also, patients receiving treatment had a higher incidence of comorbidities and complications compared to patients without treatment. Myopathy was the only high frequency condition where patients with treatment had a lower incidence than patients without treatment (IRR: 0.5; *p* < 0.05 for both Surgery Only and Surgery + Meds cohorts compared to the None cohort). The acromegaly patients in the current study shared many of the same clinical features of acromegaly patients studied in other clinical settings. Although the types of morbidity were similar, the observed prevalences were often lower than the ranges of morbidity reported elsewhere [2, 6, 7, 10, 11, 13]. For example, Davi’ et al. [14] reported that the prevalence of sleep apnea syndrome was 47 % and ranged from 39 to 56 % for patients with controlled and uncontrolled disease. At most, the prevalence in our population was 29.6 % (48/162) for patients in the surgery only cohort. Different study designs and/or coding errors in the claims may explain the varying prevalence estimates in the different studies.

As with all studies relying on retrospective administrative claims data, there are limits to how well claims can capture an individual’s medical history. Claims data are collected for the purpose of payment. As a result, the presence or absence of a diagnosis code on a medical claim does not confirm or deny actual disease. Likewise, procedure codes may be missing from inpatient claims, thus the data related to surgeries and other inpatient procedures may be incomplete.

A similar limitation also applies to claims for prescribed medications. Presence of a claim for a dispensed medication does not guarantee that the medication was taken and taken as prescribed. Additionally, patients could have received medication samples from the physician’s office, filled a prescription outside the health care pharmacy system, or received medication during a clinical trial, all of which are not captured in the claims. As a result, cohort and subgroup assignments may not correctly reflect patients’ actual treatment experiences.

Although the treatment status cohorts may be limited by coding errors and missing clinical information in the claims, they are measures of treated and untreated disease during the study’s observation period and may be viewed as an available proxy of active disease status. Unfortunately, disease control, which may be viewed as more clinically relevant, does not have an available proxy in the claims. In this managed care population, 55 % of patients without treatment had a significantly lower incidence of most major comorbidities than patients with treatment, including hypertension, diabetes, valvular heart disease, and cardiac arrhythmias. The lower incidence of comorbidities and complications among patients with no treatment may have resulted from patients receiving a surgical cure or entering remission prior to the observation period. While the higher incidence of comorbidities and complications among patients with treatment may have resulted, in part, from some patients obtaining inadequate disease management and/or poor disease control.

Of the 28 % of patients with evidence of an acromegaly-related medication, a greater proportion of patients had claims for octreotide, cabergoline, and bromocriptine than lanreotide and pegvisomant. Pegvisomant is a newer GH antagonist that requires daily injection. It is indicated as a second-line treatment in evolutive acromegaly and administered to patients showing resistance to octreotide [1, 15]. Although expensive, for some patients with overly active acromegaly, it is the only way to control the disease [16].

The use of cabergoline and bromocriptine as a primary choice during the study period of 2002 through 2010 is likely due to their oral, rather than injectable, formulations. However, bromocriptine, even at high doses, is less efficacious than cabergoline, and cabergoline monotherapy is only efficacious in reducing serum GH below 2 μg/L in about 30 % of patients [17]. Comparatively, the efficacy of pegvisomant ranged from about 75 to 97 %, octreotide from about 65 to 79 %, and lanreotide from about 50 to 76 % for a variety of efficacy measures including, but not limited to, reducing serum GH, normalizing IGF-1, and reducing tumor size. As for octreotide acetate and octreotide LAR, studies have reported that the efficacies of the two formulations are similar. More than half (52 %) of patients in the octreotide acetate subgroup started octreotide LAR sometime during the follow-up period, which is consistent with current treatment guidelines. Current research on the long-term health risks of these medications in acromegaly patients is limited. A lack of familiarity with these issues by non-specialized clinicians may also help explain this finding. Future research substantiating the level of risk is recommended.

In addition to the limitations described above, additional limitations must be considered when interpreting the study results. First, the data used for this study came from a managed care population and may not be generalizable to other health plan populations. The plans used for analysis, however, are discounted fee-for-service, IPA-network plans and not capitated or gatekeeper models. Therefore, the results from this study are primarily applicable to populations which receive their care through similar delivery systems. The plans used for analysis included a wide geographic distribution across the United States, and therefore provide the capability for generalization to comparable managed care populations on a national level.

Second, the inherent challenges of studying acromegaly in an administrative claims database included limited availability of clinical information and laboratory results needed to accurately identify patients with acromegaly, examine the use of treatment monitoring, determine the current disease status as active or inactive, and assess whether the treated disease was controlled or uncontrolled.

Third, despite the large denominator and multiple study years in the underlying databases, sample sizes in some table cells were small, which limited the opportunity to match or adjust for patient differences in a consistent manner across key outcomes.

Acromegaly is challenging to diagnose and there are no means for accurately identifying patients with acromegaly in claims data alone; however, the inclusion of a retrospective chart review may be an option for validating the disease status, progression, and clinical outcomes observed in the present study.

In the course of this claims study, substantial emphasis was placed on multiple pathways to case identification. However, no medical chart abstraction was conducted to validate accurate case definition. The observation that 55 % of acromegaly patients had no evidence of acromegaly treatment was an unexpected finding. With the exception of the small proportion of patients for whom surgery is curative, we expected that more patients would have received pharmacological treatment for acromegaly. Future research should include a medical chart review to validate the patient identification algorithm used in this claims database study and to further study real-world comorbidities and treatment patterns in patients with acromegaly.

## Conclusions

This study examined the real-world comorbidities and treatment of 949 acromegaly patients in two large US administrative claims databases. In this study, patients with acromegaly were found to have a high level of serious comorbidities (hypertension, diabetes, valvular heart disease, and cardiac dysrhythmia/arrhythmia). The incidence of comorbidities was highest among patients with acromegaly-related treatment, which may have resulted, in part, from inadequate disease management and/or poor disease control. In addition, over half of the patients received no treatment for acromegaly (neither surgery nor medication) and only 28 % received an acromegaly-related medication during the study period. Given that cure rates with surgery alone are relatively low for macroadenomas, the most common source of GH excess, a greater use of medical therapy was expected. However, some patients may have received a curative surgery prior to the observation period, which may have reduced the use of other acromegaly-related treatments during the study period.

Additional research, including medical chart review and collection of data on GH and IGF-1 laboratory results, is warranted to further understand the factors associated with diagnosis and the potential under-treatment of patients with acromegaly. Furthermore, knowing the many clinical features of acromegaly and the side effects of its treatment may facilitate earlier diagnosis and treatment, and appropriate follow-up care, which may avoid or at least minimize the life-long consequences of uncontrolled disease.

## Appendix

See Tables 5 and 6.

**Table 5:** CPT and ICD-9-CM procedure codes for commonly used acromegaly procedures

Code type	Code	Description
CPT	61546	Craniotomy for hypophysectomy or excision of pituitary tumor, intracranial approach
	61548	Hypophysectomy or excision of pituitary tumor, transnasal or transseptal approach, nonstereotactic
	62165	Neuroendoscopy, intracranial; with excision of pituitary tumor, transnasal or trans-sphenoidal approach
	77371	Radiation treatment delivery, stereotactic radiosurgery (SRS), complete course of treatment of cranial lesion(s) consisting of 1 session; multi-source Cobalt 60 based
	77372	Radiation treatment delivery, stereotactic radiosurgery (SRS), complete course of treatment of cranial lesion(s) consisting of 1 session; linear accelerator based
	77432	Stereotactic radiation treatment management of cranial lesion(s) (complete course of treatment consisting of 1 session)
ICD-9 proc	07.61	Partial excision of pituitary gland, transfrontal approach
	07.62	Partial excision of pituitary gland, transsphenoidal approach
	07.63	Partial excision of pituitary gland, unspecified approach
	07.64	Total excision of pituitary gland, transfrontal approach
	07.65	Total excision of pituitary gland, transsphenoidal approach
	07.68	Total excision of pituitary gland, other specified approach
	07.69	Total excision of pituitary gland, unspecified approach
	92.30	Stereotactic radiosurgery, not otherwise specified
	92.39	Stereotactic radiosurgery, not elsewhere classified

**Table 6:** ICD-9-CM codes for acromegaly-related comorbidities and complications

Conditions	Codes
Local effects	
Visual-field defects	368.4x
Hypopituitarism	253.2, 253.7
Musculoskeletal	
Osteoarthritis	715.xx
Arthropathy/arthralgia/synovitis	713.x, 716.4x–716.5x, 716.8x–716.9x, 719.4x, 727.0x
Kyphosis and scoliosis	737.1x, 737.3x–737.9
Vertebral fracture	733.13, 805.xx–806.xx
Carpal tunnel syndrome	354.0
Myopathy/myalgia	359.5–359.9, 729.1
Cardiovascular	
Hypertension	362.11, 401.xx–405.xx, 642.0x–642.2x, 642.7x
Cardiomyopathy	425.4, 425.7–425.9
Cardiac hypertrophy	429.3
Heart failure	402.01, 402.11, 402.91, 404.01. 404.03, 404.11, 404.13, 404.91, 404.93, 428.xx
Valvular heart disease	424.xx
Cardiac dysrhythmia/arrhythmia	427.xx
Respiratory	
Nasal polyps	471.x
Sleep apnea (obstructive and central)	327.20–327.21, 327.23, 327.26–327.29, 780.51, 780.53, 780.57
Narcolepsy	347.xx
Endocrine/metabolic	
Diabetes (incl. impaired glucose tolerance)	249.xx–250.xx, 357.2, 362.0x, 366.41, 648.0x, 790.2x, 996.57, V45.85, V53.91, V58.67
Galactorrhea	611.6, 676.6x
Menstrual abnormality	626.x, 627.0–627.1
Impaired libido/impotence	302.72, 607.84, 799.81
Other	
Colon polyp	211.3
Hyperhydrosis	705.2x, 780.8

Modified from Cordero et al. [11]
